# How a fungus shapes biotechnology: 100 years of *Aspergillus niger* research

**DOI:** 10.1186/s40694-018-0054-5

**Published:** 2018-05-24

**Authors:** Timothy C. Cairns, Corrado Nai, Vera Meyer

**Affiliations:** Department of Applied and Molecular Microbiology, Institute of Biotechnology, Technische Universität Berlin, Gustav-Meyer-Allee 25, 13355 Berlin, Germany

**Keywords:** *Aspergillus niger*, Biotechnology, Industrial biology, Systems biology, Genome editing, Citric acid

## Abstract

In 1917, a food chemist named James Currie made a promising discovery: any strain of the filamentous mould *Aspergillus niger* would produce high concentrations of citric acid when grown in sugar medium. This tricarboxylic acid, which we now know is an intermediate of the Krebs cycle, had previously been extracted from citrus fruits for applications in food and beverage production. Two years after Currie’s discovery, industrial-level production using *A. niger* began, the biochemical fermentation industry started to flourish, and industrial biotechnology was born. A century later, citric acid production using this mould is a multi-billion dollar industry, with *A. niger* additionally producing a diverse range of proteins, enzymes and secondary metabolites. In this review, we assess main developments in the field of *A. niger* biology over the last 100 years and highlight scientific breakthroughs and discoveries which were influential for both basic and applied fungal research in and outside the *A. niger* community. We give special focus to two developments of the last decade: systems biology and genome editing. We also summarize the current international *A. niger* research community, and end by speculating on the future of fundamental research on this fascinating fungus and its exploitation in industrial biotechnology.

**Electronic supplementary material:**

The online version of this article (10.1186/s40694-018-0054-5) contains supplementary material, which is available to authorized users.

## Introduction

For millennia, humanity has practiced rudimental forms of biotechnology: by fermenting starch and sugars present in grains and fruits, ancient civilizations were able to produce bread, beer, wine, and other alcoholic beverages. Prior to the late nineteenth and early twentieth century, these processes were conducted without knowledge of the underlying biological events. Now, brewery and wine making is a well-understood, controlled industrial process. Similarly, in just a century, industrial biotechnology has changed dramatically and flourished, from initial proof-of-principle experimentation in Erlenmeyer flasks, to a multibillion dollar industry producing megatons of useful molecules [1]. Fungal biotechnology is undoubtedly a major contributor and driver of this success. As just one example, the estimated market volume for plant-degrading enzymes from filamentous fungi in 2016 was €4.7 billion, which was expected to reach up to €10 billion within the next decade [2]. In this celebratory historical overview, we outline some of the crucial advances for the filamentous mould *Aspergillus niger* since the very first biotechnological experiments using this fungus 100 years ago.

## 100 years ago: industrial biotech is born

In contrast to what most people might think, citric acid is not—or not anymore—isolated from citrus fruits, but is industrially produced by the filamentous fungus *A. niger*. The process was pioneered by James Currie, a food chemist, who 100 years ago published a study describing the superior properties of *A. niger* for the industrial production of the acid [3]. In particular, Currie showed the necessary growth medium for citric acid biosynthesis, and the ability of the fungus to grow at low pH (2.5–3.5), while still being able to produce high amounts of the metabolite. Moreover, this work demonstrated the direct correlation between amount of substrate in the medium and amount of product, laying the basis for modern-day industrial fermentation of citric acid [3]. In contrast to other species of fungi that had been reported to produce citric acid by 1917, every single strain of *A. niger* that Currie tested could efficiently produce this molecule when grown in sugar solutions. Two years later, the American company Pfizer made a pilot plant for biochemical production of citric acid, and by the mid 1920s, production using *A. niger* fermentation far outweighed extraction from citrus fruits [4].

The citric acid cycle was comprehensively determined over the next several decades, resulting in the award of the Nobel Prize to Hans Krebs and Fritz Lipmann in 1953. The first and final reaction in the cycle involves the formation of citrate from oxaloacetate, acetyl-CoA, and water, by a citrate synthase, ultimately generating chemical energy in the form of adenosine triphosphate (ATP). The objective of industrial microbiologists, including James Currie, was to exploit this cycle, and indeed many other metabolic pathways, to ferment useful molecules.

Although there are variations in fermentation techniques, in general, industrial production of citric acid requires aerobic, submerged growth of *A. niger* in a sugar solution, which is usually derived from inexpensive sources, such as molasses, corn steep liquor, or hydrolysed corn starch, amongst others. After fermentation, *A. niger* is physically removed, usually by filtration, and citric acid is isolated by precipitation of the fermentation mix with calcium hydroxide (lime) to generate calcium citrate salt. Subsequent treatment with sulphuric acid yields the citric acid product.

The widespread applications of citric acid are shown by current figures regarding this metabolite: in 2007, worldwide production was estimated at 1.6 million tons, with an estimated value of $2.6 billion in 2014 and predicted to rise to $3.6 billion by 2020 [1, 5]. As a weak acid, it can be used as an antioxidant, preservative, acidulant, pH-regulator, or flavour in food and beverages, as well as comparable applications in the pharmaceutical and cosmetics industries. Citric acid is currently predominantly produced in China, which accounts for approximately 60% of global production [1]. However, *A. niger* industrial applications are not just limited to the production of citric acid; as a prolific secretor, numerous industrially relevant enzymes and other molecules are produced by this fungus. Below, we summarize some of the key developments in the field over the last century.

## A historical snapshot of *A. niger* research

**Fig. 1:**
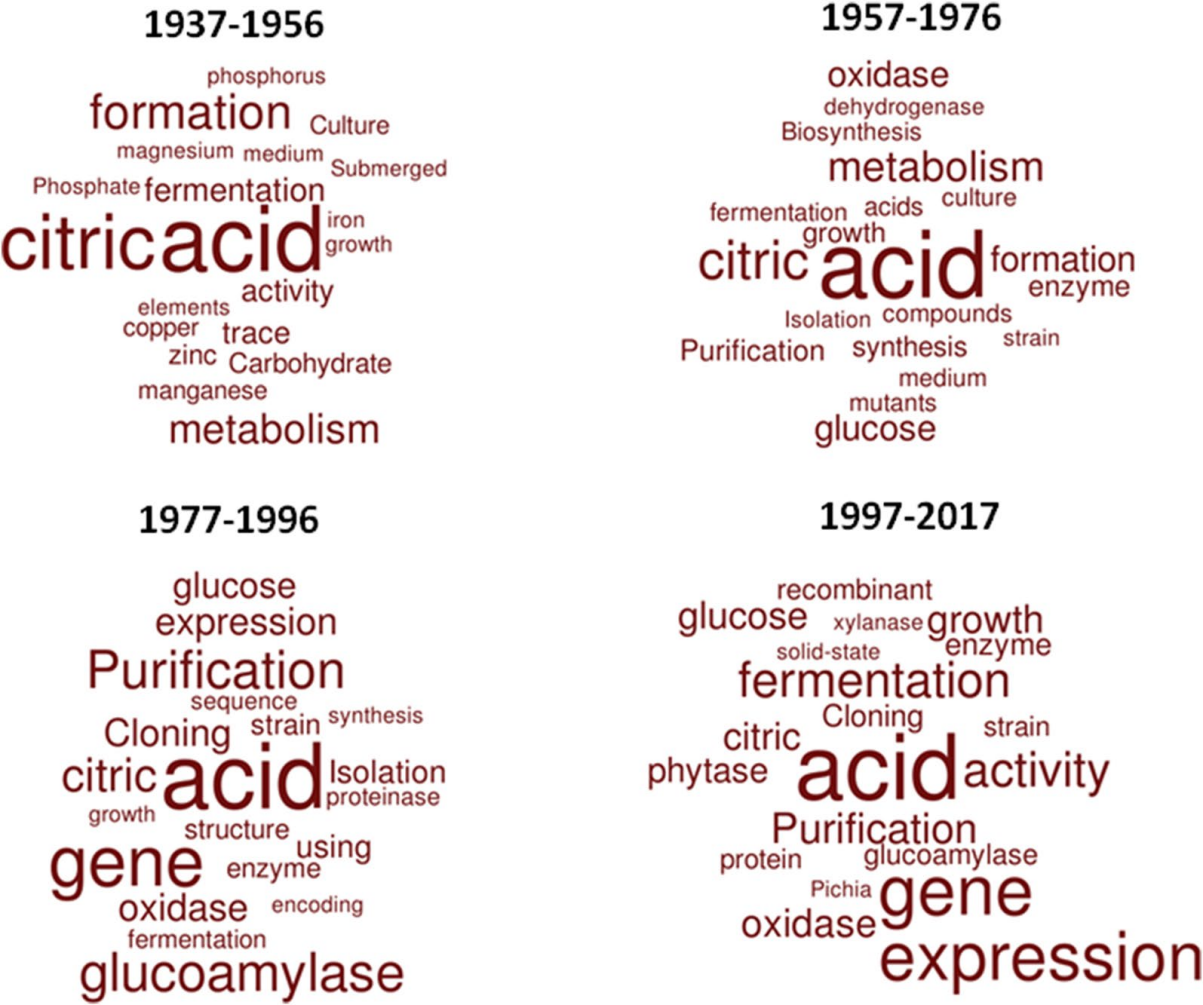
A historical snapshot of *A. niger* research. The PubMed database was interrogated for any publication that contained ‘*Aspergillus niger*’ in the title. Publication titles were assigned to periods of 20 years. As 1917–1936 only returned 7 manuscripts, this period was omitted. Word clouds were generated online (https://worditout.com/word-cloud/create), and the top 20 words, not including ‘*Aspergillus niger*’ or non-technical terms (e.g. prepositions etc.), are depicted. Size of each word is proportional to relative frequency amongst all the titles in that period. Retrieved articles for each period: 112 (1937–1956), 481 (1957–1976), 642 (1977–1996), 1789 (1997–2017)

The fundamental and applied scientific discoveries using *A. niger* over the last 100 years are extremely diverse. As Currie wrote in 1917: ‘Few concise statements can be made concerning the metabolism of an organism capable of producing such a variety of chemical transformations as *Aspergillus niger*’ [3]. Nevertheless, some trends regarding the history of the *A. niger* research field can, in general terms, be deciphered. We conducted a survey of the *A. niger* literature since Currie’s seminal study by interrogating the PubMed database [6] for any publication containing ‘*Aspergillus niger*’ in the title. The resulting articles (> 3000, see Additional file 1: Table S1) were divided into five 20-year periods based on their publication date, and the 20 most common words from the available titles during each time period were visualized as word clouds (Fig. 1). Although querying the PubMed database for ‘*Aspergillus niger*’ in abstract and keywords resulted in more returned manuscripts (> 8700 hits), we decided to limit our word-cloud analysis specifically to titles. We applied this restriction as searching among manuscript abstracts returned a majority of hits where researchers used *A. niger* in simple growth assays to validate efficacy of putative antifungals. While of interest, these research efforts (which were extremely common from 1977 onwards) are not, specifically, interested in *A. niger* biology per se.

**Fig. 2:**
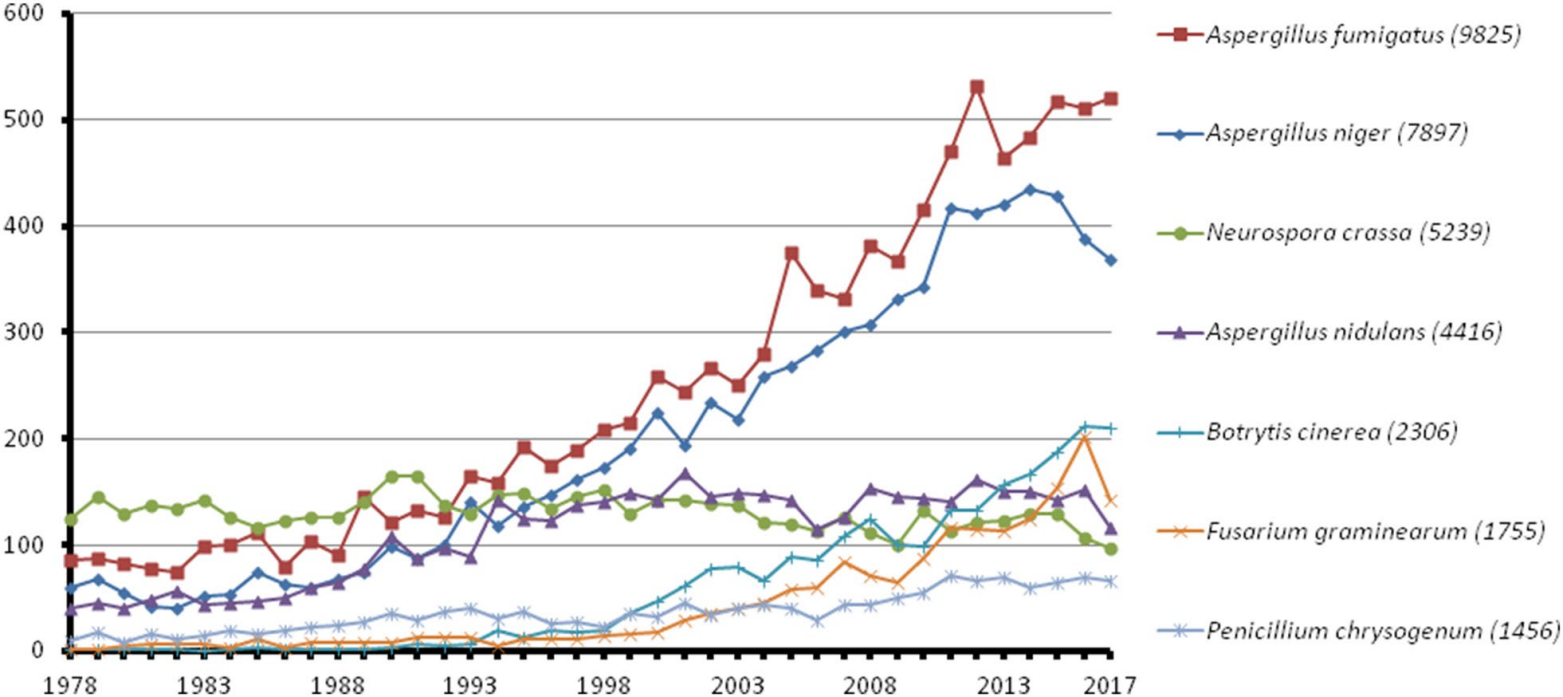
Total number or paper published on PubMed for selected filamentous fungi over the last 40 years. The literature database was queried for title, abstract and keywords with the full species name (in brackets: total results for 1978–2017). As comparison: *Saccharomyces cerevisiae* gave more than 110,000 hits with around 2000–4000 hits/year over the last 25 years (Additional file 2: Table s2)

Our analysis of 100 years of *A. niger* publications indicated, unsurprisingly, that ‘citric acid’ and ‘fermentation’ were amongst the most common returned words in titles from every period (Fig. 1). Clearly, Currie’s discovery of conditions for maximizing citric acid production [3] was indeed a biotechnological revolution, and one that would constitute a major focus of research over the next century. Indeed, *A. niger* research has rapidly grown over the past 40 years (Fig. 2). Although by no means exhaustive, distinct historical trends become apparent from our analysis (Fig. 1).

### The foundations of *A. niger* research

From the period 1937–1956, *A. niger* researchers were predominantly concerned with the impact of micro/macro nutrients in cultivation media and growth parameters for optimized citric acid fermentation (Fig. 1, [7, 8]). Most studies specifically utilized submerged culture, which obviously reflects the need to control growth, morphology, and, ultimately, citric acid production during fermentation (Additional file 1: Table S1 and [9]). After the award of Hermann Muller’s Nobel Prize in 1946 for work on mutations via X-ray irradiation, the late 1940s and early 1950s saw the first mutagenesis studies in biological sciences deployed by the *A. niger* community in order to generate strains with improved citric acid yields [10]. The number of UV, X-ray, and chemical mutagenesis efforts increased from 1957 to 1976, with most focusing on enhancing citric acid production (e.g. [11] and Fig. 1).

Notably, the period from 1957 to 1976 saw a paradigm shift amongst the *A. niger* biotechnological research community, with the widespread realization that this fungus was not exclusively applicable for citric acid fermentation, but was also a prolific producer of useful enzymes (Fig. 1). A rapid growth of studies, facilitated by technological advances in chromatography, conducted purification and enzymatic analysis of diverse range of *A. niger* proteins, including various oxidases, dehydrogenases, hydrolases, cellulases, and pectinases, amongst others (Fig. 1, [12–16]). These discoveries were not only useful for industrial production of enzymes, but also significantly contributed to fundamental understanding of enzyme function. One notable pair of isoenzymes first purified in the late 1960s and early 1970s was *A. niger* glucoamylases [17, 18]. These discoveries would eventually lead to the widespread application of *A. niger* glucoamylases in the fermentation, food, and beverage industries, where these enzymes catalyse the saccharification of partially processed starch to glucose. Indeed, glucoamylase research features heavily in publications from 1977 to present day (Fig. 1). Interestingly, a recent business report showed that the glycoamylase market played a significant role for the success of leading multinational biotech companies, including AB Enzymes, Amano Enzyme, DSM, Genencor, Novozymes, and Verenium [19].

Outside the field of industrial microbiology, there was early interest in the 1970s in the clinical spectrum of disease caused by the *Aspergillus* genus, with authors first associating *A. niger* spore inhalation with the onset of asthma [20]. The serious threat of fungal disease both with regards to human health and crop destruction is now much better understood [21]. Indeed, fungal infections affect and estimated 1.2 billion people globally, resulting in approximately 1.5–2 million deaths per year. As a consequence of limited antifungal therapeutic options and timely diagnostics, mortality rates can be extremely high, reaching up to 90% in case of immunocompromised patient and/or drug-resistant strains causing invasive aspergillosis or other systemic mycoses [2]. Indeed, the fungicide market to control fungal growth in agriculture and medicine was estimated at €10 billion in 2014 [2].

### The dawn of molecular biology and the first *A. niger* transformations

A revolution in biotechnology occurred between 1977 and 1996 with the advent of molecular biology. These technological developments and rapid discoveries in *A. niger* molecular biology and genetics are reflected by a heavy community-wide focus on genes, cloning, and sequence analyses (Fig. 1). Arguably the most fundamental technique for molecular analyses of any organism is the ability to transform exogenous DNA into target cells and integrate them into recipient genomes. In a seminal study, Peter Punt and Cees van den Hondel utilised a hygromycin resistance gene from *Escherichia coli*, encoding a phosphotransferase, as a dominant selectable marker in *A. niger* and *A. nidulans* [22]. In addition to becoming one of the most commonly used dominant selectable markers in fungal transformation, this work pioneered plasmid mediated cassette integration in filamentous fungal genomes using the vector pAN7-1. Moreover, the authors further validated the use of the *A. nidulans* glyceraldehyde-3-phosphate dehydrogenase (*gpd*) promoter, and the *trpC* terminator. In an alternative approach, other studies optimized a homologous transformation system using the orotidine 5′-phosphate decarboxylase gene *pyrG* [23, 24]. This recyclable auxotrophic marker would ultimately facilitate several hundred studies of *A. niger* gene function, and is still used today, most obviously as a transformation system, but also as a convenient locus for cassette integration during heterologous and homologous gene expression studies [25].

Introduction of such molecular studies began to enable industrial microbiologists to obviate a diverse range of technical challenges when working with filamentous fungi. As just one example, heterologous expression of a porcine pancreatic phospholipase gene in *A. niger* by the lab of David Archer initially did not produce detectable protein, which was revealed to be due to degradation of this enzyme by intracellular and extracellular *A. niger* proteases [26]. The authors therefore used PCR and restriction endonuclease cloning approaches, which were cutting-edge at the time (and indeed still practiced in most molecular laboratories today), to express a prophospholipase-glucoamylase fusion protein in a protease-deficient *A. niger* strain. This recombinant approach enabled secreted concentrations of fusion protein at 10 μg/mL.

The utilisation for *A. niger* for production of a diverse range of enzymes also continued between 1977 and 1996, as exemplified by numerous efforts during this period for the fermentation and purification of proteases (Fig. 1). Fungal proteases are active at a broad range of abiotic conditions (e.g. pH, temperature) and consequently are now applied in food, laundry detergent, and pharmaceutical processes, amongst others (reviewed in [27]).

### The last 20 years: rapid developments in the *A. niger* research field

The development of the *A. niger* molecular toolkit accelerated from 1997 to 2017. Notable milestones include the generation of nonhomologous end joining (NHEJ) mutants in a collaborative effort between our lab and the lab of Arthur Ram [28], which enable increased targeting efficiency of exogenous DNA cassettes with recipient genomes during fungal transformation. Filamentous fungal NHEJ mutants were first described in the model organism *Neurospora crassa* in 2004 [29] and the drastic increase in cassette targeting rates (up to 100% of transformed fungal cells) led to the rapid application of this tool by researchers in the *Aspergillus* genus, including *A. niger* [28, 30].

The application of *A. niger* as a cell factory for useful enzymes continued to rapidly expand between 1997 and 2017 (Fig. 1). Phytases, for example, were first marketed in 1991, and are used to improve the nutritional content of animal feed by generating inorganic phosphorus from phytic acid, which is the major form of organic phosphorus in plant seed [31]. The biotechnological production market of phytases is estimated to be over €150 million per year, with *A. niger* one of the most commonly used microorganisms [32].

In addition to homologous or heterologous production of single industrially relevant proteins, *A. niger* and other Aspergilli have been increasingly harnessed for synthesis of diverse enzyme mixtures over the last decade. One critical application of such enzyme cocktails is the degradation of plant polysaccharides, whereby cellulose, hemicellulose, and pectin can be broken down into oligosaccharides and monosaccharides and the number of *A. niger* genes predicted to encode proteins capable of plant biomass degradation is over 170 [33]. Moreover, the transcription factors that regulate these genes are being rapidly elucidated, for example the amylolytic regulator AmyR (the first regulator identified in *A. niger*) [34], pectinolytic regulator RhaR [35], and hemi-cellulolytic regulator XlnR [36, 37], amongst several others (reviewed in [33]). Integrating knowledge of transcription factor networks with a comprehensive understanding of upstream molecular sensors and signalling cascades may enable the engineering of *A. niger* isolates with increased plant biomass degradation capabilities. The impact of such microbial cell factories will enable the renewable generation of starting material for production of biofuels and other industrial processes from plant material. Consequently, future *A. niger* strains may enable transition from our current fossil-based economy to a bio-based economy, whereby future fuel is generated from renewable resources.

In general, between 1996 and present day, much progress has been made for enzyme expression using *A. niger*, with higher titres of secreted proteins increasingly possible (e.g. 30 g/L for glucoamylase is common) (reviewed in [38, 39]). These advances have been achieved by several now routine approaches, including codon optimisation of non-fungal genes, and, in some instances, use of a fusion carrier protein [38]. However, a significant bottleneck that inhibits maximum yield of secreted proteins is an incomplete understanding of filamentous fungal secretion. In the model postulated by Taheri-Talesh using *A. nidulans* [40], secretion and polar growth are physically coupled at the hyphal tip (first showed in *A. niger* by Han Wösten et al. in 1991 [41]). Currently, however, the underlying mechanisms of fungal protein secretion are not understood as an integrated system, with numerous outstanding questions hampering rational strain engineering. For example, by investigating secretome in concentric zones of *A. niger* colonies it has been observed that secretion and growth can be uncoupled [42]. How can this phenomenon be further exploited to further increase secretion yields without affecting hyphal tip growth? What molecular signals and regulators control and limit protein secretion? We speculate that delivering a systems-level understanding of the molecular and cellular mechanisms that underpin fungal secretion will one of the major research goals over the next 20 years.

Several studies over the past decade have interrogated the effect of filamentous microscopic and macroscopic morphologies on secretion during industrial applications. For example, *A. niger* hyperbranching phenotypes have been generated to study the underlying morphogenetic gene network controlling polar growth, and, elsewhere, secretion or the macromorphology of *A. niger* has been modified by the addition of micro-particles to submerged media [43, 44]. These genetic or microbiological approaches offer increasingly accurate control of hyphal branch length, enabling optimization of fermentation culture viscosity, and also minimising *A. niger* sensitivity to shear stress. Intriguingly, a promising avenue of research for maximising industrial protein titres comes from analyses of secretion in *A. oryzae*, which has conclusively demonstrated that secretion also occurs at septal junctions [45, 46]. This might conceivably be harnessed by industrial microbiologists as a secondary secretion route to maximize secretion of useful enzymes in Aspergillus species, including *A. niger*. Another level of complexity behind *A. niger* secretion was uncovered by pioneering studies of the labs of Han Wösten, Arthur Ram and Cees van den Hondel: the discovery of heterogeneity of fungal secretion on the cell [47], hyphal [48] and colony level [49]. This work laid the conceptual framework for follow-up studies on population heterogeneity in model and industrial Aspergilli [50–52].

Outside the field of industrial microbiology, one alarming observation is that the last century of *A. niger* research spans the discovery of penicillin, for which Alexander Fleming, Ernst Chain, and Howard Florey were awarded the Nobel Prize in 1945, and the subsequent global emergence of drug resistant pathogenic microbes. Currently, as the incidence of drug resistance increases, the number of chemical compounds approved for use in agriculture or the clinic is decreasing [53]. Given that microbial secondary metabolites are a rich source of novel bioactive molecules [54], the focus of our lab and others over the last 5 years has been to establish *A. niger* as an industrial platform strain for drug discovery and natural product production. This objective is based on the assumption that the high intracellular glycolytic flux towards citric acid (and amino acids derived thereof) can be exploited and redirected into non-ribosomal peptide synthesis. Recent work has demonstrated we could indeed genetically engineer *A. niger* to heterologously overexpress a non-ribosomal peptide synthetase (NRPS) from *Fusarium* spp. in several g/L amounts [55]. This proof of principle experiment, which utilized the highly optimised and titratable synthetic Tet-on gene switch [25] to produce the antimicrobial cyclohexadepsipeptide enniatin, will hopefully pave the way for future production of multiple secondary metabolites. Indeed, more recently, we could generate *A. niger* isolates expressing truncated enniatin NRPS enzymes or with key domains positionally exchanged, to generate new-to-nature molecules at high titers (e.g. 1.3 g/L) [56]. Excitingly, some of these new molecules demonstrated enhanced antiparasitic activity when compared to existing drugs [56]. Given the high diversity of fungal secondary metabolite genes, and their frequent transcriptional silence under laboratory conditions, expression using *A. niger* as a heterologous host, and the molecular approaches validated by these two studies, hold great promise for discovery of new chemical leads for compound development in agriculture and the pharmaceutical industry. This is further supported by recent work that has applied viral DNA sequences (encoding, for example, the 2A peptide), in order to enable polycistronic gene expression in *A. niger* [57, 58]. These studies provide proof of principle that complex secondary metabolites, which require multiple enzymes for their biosynthesis, can be produced by polycistronic gene switches in *A. niger*.

To summarize, one century after James Currie’s ground-breaking work on biotechnological citric acid production, his assessment still generally holds true: it is difficult to make comprehensive statements about the production capabilities of *A. niger* owing to its metabolic versatility, and the many as-yet undisclosed metabolic pathways. However, the dawn of molecular biology, and recent breakthroughs in synthetic biology for *A. niger*, have ultimately engineered a multipurpose cell factory out of a citric acid producer. *A. niger* is the most versatile filamentous fungal platform strain which can now be exploited to produce acids, proteins, enzymes, and medicinal drugs (Table 1). Two developments that occurred over the last decade promise to open entirely new avenues of scientific study using *A. niger*: genome sequencing and the introduction of genome editing. The following sections look at these two developments in more detail.

**Table 1:** Selection of (multi)national companies exploiting *A. niger* for the production of important industrial compounds. Modified after Fiedler et al. 2013 [79]

Company	Headquarter	Products
Adcuram	Germany	Citric acid
AB Enzymes	Germany	Glucoamylase
ADM	USA	Citric acid
Agennix	Germany	Lactoferrin
Amano Enzyme Co. Ltd.	Japan	β-Galactosidase, Glucoamylase, Glucose oxidase, Hemicellulase, Proteases
Anhui BBCA Biochemical	China	Citric acid
BASF	Germany	Hemicellulase, Phytase
Biocon	India	Cellulase, Hemicellulase, Pectinase
Cangzhou Kangzhuang Chemical	China	Glucoamylase
Cargill	USA	Citric acid
Christian Hansen	Denmark	Chymosin
COFCO	China	Citric acid
DSM	The Netherlands	Arabinase, Asparaginase, Catalase, Cellulase, β-Galactosidase, Glucoamylase, Glucose oxidase, Hemicellulase, Lactoferrin, Lipase, Pectinase, Phytase, Proteases, Xylanase
Dupont IB	The Netherlands	Catalase, β-Galactosidase, Glucoamylase, Glucose oxidase, Hemicellulase, Lipase, Pectinase
Dyadic	USA	Cellulase, Glucoamylase, Glucose oxidase
Gadot Biochemical Industries	Israel	Citric acid
Genencor INT	USA	Cellulase, Hemicellulase, β-Galactosidase
Haihang Industry	China	Cellulase
Iwata Chemical Co. Ltd	Japan	Citric acid
Jungbunzlauer	Switzerland	Citric acid
Mitsubishi Foods Co. Ltd.	Japan	Proteases
Megazyme	USA	Catalase, Inulinase, Glucosidase
Novozymes	Denmark	Asparaginase, Catalase, β-Galactosidase, Glucoamylase, Hemicellulase, Lipase, Pectinase, Phytase, Proteases
Shandong Longda Bio-Products	China	Glucoamylase, Pectinase
Shin Nihon Chemical Co. Ltd.	Japan	Arabinase, Catalase, Cellulase, β-Galactosidase, Hemicellulase, Proteases
Tate & Lyle	UK	Citric acid
Verenium	USA	Glucoamylase, Proteases

## 2007: the genome sequence of *Aspergillus niger* is released

The first filamentous fungal genome to be published was that of the model ascomycete *Neurospora crassa* in 2003, 160 years after the discovery of this species in a Paris bakery [59]. Several hallmarks of fungal genomes were reported in this ground-breaking draft, in particular (1) contiguous gene clusters for secondary metabolite biosynthesis; (2) defence from parasitic mobile genetic elements via repeat induced point mutation; (3) variations in telomeric gene content when compared to telomere-distal chromosome regions; (4) and the presence of two putative RNA silencing pathways, amongst others [59]. It was in this context that the first *A. niger* genome was released in 2007 [60], by which time three Aspergillus genomes were also publicly available: *A. nidulans* [61], *A. oryzae* [62], and *A. fumigatus* [63]. These genomes were representative of the broad utilities and challenges posed by the *Aspergillus* genus: model organism, food producer, and human-infecting fungus, respectively. The publication of the *A. niger* genome was thus the first, and, arguably, the most important, industrial *Aspergillus* genome to be sequenced. For this draft assembly, Herman Pel and colleagues used the enzyme producing isolate CBS 513.88, which is a derivative of *A. niger* NRRL 3122, a strain generated by classical mutagenesis for glucoamylase A production [60]. This was therefore the first global analyses of the *A. niger* genome repertoire that had been harnessed in industrial applications for many decades, and remains one of most comprehensively annotated genome resource for the *A. niger* community.

Numerous explanations of the suitability of *A. niger* for industrial applications were identified from the estimated 34 Mb genome, with its estimated 14,165 coding genes. For example, the authors predicted various gene duplication events that may have led to expansion of genes necessary for the production of the citrate precursor oxaloacetate [60], an observation that explains the remarkable capability for citric acid production by *A. niger*. Indeed, this hypothesis has been supported by a very recent comprehensive comparative genomic analysis of black aspergilli in a community effort led by Ronald de Vries [64], which was published exactly 10 years after the public release of *A. niger* genome sequence. With regards to the nutritional versatility of *A. niger*, genes encoding putative nutrient transporters were enriched in *A. niger* when compared to *A. nidulans* or *A. fumigatus*. These genes were presumed to enable uptake or sensing of diverse carbon and nitrogen sources [60].

In the context of novel bioactive molecule discovery, numerous putative secondary metabolite loci were identified based on the presence of either a polyketide synthase (PKS) or NRPS encoding gene [60]. Intriguingly, the vast majority of these clusters lacked either an experimentally verified or predicted biosynthetic product, thus indicating the potential for novel pharmaceutical discovery using *A. niger* and other Aspergilli [65, 66]. Indeed, these observations have been corroborated by recent comparative genomic analyses of the *Aspergillus* genus [64], which indicate that *A. niger* CBS 513.88 has 57 predicted secondary metabolite clusters, whilst another study predicts 81 secondary metabolite clusters [67], the highest of all Aspergillus genomes analyzed so far. The latter study follows an extensive manual annotation approach and likely more precisely predicts the actual secondary metabolite repertoire of *A. niger*. Taken together, these exemplar discoveries highlight how the *A. niger* draft genome provided the first global explanations for the many industrially relevant phenotypes of this organism, and facilitated a new era of forward genetics in this species. Moreover, in the immediate aftermath of this revolutionary resource for the *A. niger* community, comparative genomic analyses amongst the aspergilli would also redefine species concepts [68], interrogate sexual compatibility [60], and the nature of fungal virulence [69], amongst other critical developments (reviewed in [70]).

DNA sequencing technology and analyses are now sufficiently accurate and high throughput to be routinely applied to answer a diverse range of fundamental research questions in *A. niger*. One recent and notable example is the so called bulk segregant analyses developed by Arthur Ram’s lab, which was used to identify a single nucleotide polymorphism (SNP) responsible for a nonacidifying phenotype of a UV-mutated isolate [71]. In the bulk segregant approach, the *A. niger* parasexual cycle is used to cross a wild-type strain with a mutant of interest, and haploid segregants with the phenotype of interest are identified. Subsequently, DNA from these segregants and parental isolates are sequenced to identify SNPs. Amongst these isolates, the SNP that is conserved in all segregants, yet absent in the wild-type isolate, is responsible for the mutant phenotype. Fascinatingly, they demonstrated that the nonacidifying mutant phenotype was due to a lack of citric acid secretion, and the SNP was located in the gene encoding the putative methyltransferase LaeA [71]. This protein is a component of the velvet complex, which regulates light responses, development, and secondary metabolism in Aspergilli [72]. Consequently, these systems genetics approaches have shed light on the link between LaeA, citric acid, and secondary metabolism in *A. niger*.

### Analyses of *A. niger* genome sequences identify several challenges that are yet to be comprehensively resolved

Several pitfalls to industrial applications of *A. niger* were also highlighted from the publication of the draft genome [60]. Unsurprisingly, numerous predicted protease encoding genes were identified, many of which contained a secretion peptide, which undoubtedly pose a significant challenge to industrial protein production. Additionally, gene clusters predicted to biosynthesize the mycotoxins fumonisin and ochratoxin A were present in CBS 513.88. Subsequent metabolomic analyses led by Jens Frisvad and his colleagues suggest up to 10% of industrially used *A. niger* isolates are able to produce these potential carcinogens [73]. In addition to potential issues with toxicity, production of unwanted secondary metabolites might confound production efforts of heterologous metabolites or new-to-nature compounds, as these molecules will be produced under similar conditions, and will likely be co-extracted during proof-of-concept of scale-up stages. However, with genome editing technology (see below), it should be possible to tackle this problem by removing mycotoxin clusters [2].

A more general problem that became apparent from the *A. niger* genome sequence, however, was that functional predictions were only possible for approximately half of the putative 14,165 putative open reading frames [60]. Subsequent release of additional *A. niger* genomes [74, 75] and the continued improvement and refinement of online genome databases and analyses portals [76–80] have not drastically increased rates of gene functional annotation. Indeed, genome mining of the acidogenic isolate ATCC 1015 [74] using the publicly available analyses portal FungiDB [79] indicates that 4491 (approximately 41%) of predicted genes encode products that are annotated as a ‘hypothetical protein’, which also lack any functional prediction based on Gene Ontology (GO) or Interpro Domains.

This genomic ‘black box’ presents several challenges for systems level understanding of *A. niger* and rational strain engineering for industrial applications. Firstly, while several thousand ‘hypothetical’ genes are transcriptionally active during a diverse range of experimental conditions, many of which model industrial processes [79], the incentive to study these genes using time and labour-intensive loss-of-function approaches is very low. This is further complicated by functional redundancy, where deletion of a single gene has no measurable impact. This problem has been partially obviated by continued molecular tool development in *A. niger*, as highly optimized, titratable, and inducible/repressible promoters have been developed [81]. These molecular tools enable expression of a gene above the native levels, leading to measurable phenotypic effects in so called ‘gain-of-function’ approaches, with the added advantage that it is possible to functionally characterize essential genes. However, these strategies are unlikely to have the necessary throughput for functional characterization of several thousand genes.

Secondly, assigning functional prediction to the genomic ‘black box’ using gene orthology is also problematic, as model (or reference) organisms can be misleading. Indeed, inferring function from unicellular yeasts, such as *Saccharomyces cerevisiae*, or other Aspergilli, such as *A. nidulans*, is at best advisory, and at worst misleading, with genes and encoded products playing different roles between organisms [2, 82]. Recent applications of genome editing in filamentous fungi arguably hold the greatest promise for rapid gene functional characterization in *A. niger* [83, 84], with the potential to lead to comprehensive, systems level understanding of this organism, or to engineer new synthetic or semi-synthetic derivatives for highly optimized industrial applications.

## A new era: genome editing with CRISPR/Cas

Actually described 30 years ago in 1987 [85], CRISPR (clustered regularly interspaced palindromic repeats) elements have been universally exploited along with associated endonucleases (for example proteins of the Cas family) for about 5 years. Early DNA sequencing of bacterial genome revealed short repetitive sequences with unknown functions in *E. coli* [85], which were then discovered across many bacterial species and two decades later revealed as an adaptive defence mechanism against bacteriophages [86]. While studying ways to reduce susceptibility of starter cultures for yoghurt production against phages, industrial biotechnologists observed that cultures previously exposed to a virus where resistant upon a second encounter with the same [86]—the reason being the specific recognition, double-strand cut and inactivation of the invading DNA by the CRISPR/Cas9 system. In a seminal 2012 paper, it was shown that the system is programmable to cut any DNA sequence with high specificity [87], which generated a watershed momentum, given the ability of the endonuclease to retain its activity in many different organisms (fungi, insects, mice, humans, plants etc.). The use of the genome editing technology in filamentous fungi has been recently reviewed [88]. While delivery of DNA encoding the components of the system (endonuclease, guide RNA), or in vitro generated components themselves, into the fungal cell remains a challenge due to the fungal cell wall, and still requires common protocols as protoplasting, different strategies have been developed to increase efficiency in species such as *A. niger* and other *Aspergillus* spp., *Alternaria alternata*, *Coprinopsis cinerea*, *Ustilago maydis*, *Trichoderma reesei*, *Neurospora crassa*, *Penicillium chrysogenum*, *Myceliophthora thermophila*, *Beauveria brassiana* (reviewed in [88]). These strategies include the use of efficient promoters like *trpC*, *gpdA* or RNA polymerase III promoters like *U6* for in vivo expression of endonuclease and/or gRNA, codon optimization of Cas9, transient expression of endonuclease, integration of endonuclease into the host genome, delivery of in vitro synthesized gRNA or purified endonuclease, and others. Also, considerations concerning the use of appropriate markers (following transformation of CRISPR/Cas components or genome editing) and lethal or unwanted (e.g. off-target) effects of the endonuclease have been addressed in filamentous fungi. For the latter, specificity of genome editing can be increased by (1) favouring host’s homology-directed repair (HDR) over non-homologous end-joining (NHEJ) pathway following DNA cut, or (2) generating DNA double-strand breaks with long overhangs by using either a modified Cas9 (nickase Cas9—a.k.a. nCas9—able to cut only one DNA strand and thus following duplexing with two distinct gRNA generating long “sticky ends”) [89].

With the proper strategy, CRISPR/Cas technology looks like a spider able to catch a couple of flies; indeed, a broad spectrum of opportunities arises for strain engineering. One recent example illustrates the reach of the technology when applied to *A. niger* and other filamentous fungi. Kuivanen et al. [90] used CRISPR/Cas technology to delete multiple genes in *A. niger* for the biotechnological production of the platform chemical galactaric acid. Derived from d-galacturonic acid, the main component of the natural polymer pectin, galactaric acid is used as precursor for Nylon and in skin-care cosmetics [90]. Although *A. niger* can hydrolyze pectin, d-galacturonic acid is also a precursor for the fungal galacturonic acid pathway, and galactaric acid can be catabolized by an unknown pathway. The authors deleted seven genes involved in catabolism of d-galacturonic acid and galactaric acid in *A. niger* using a strategy involving in vitro synthesized gRNA and plasmid-encoded Cas9. With such an engineered *A. niger* strain, the authors showed the digestion of pectin-rich biomass into galactaric acid in a single process [90].

## The *Aspergillus niger* community as of today

**Fig. 3:**
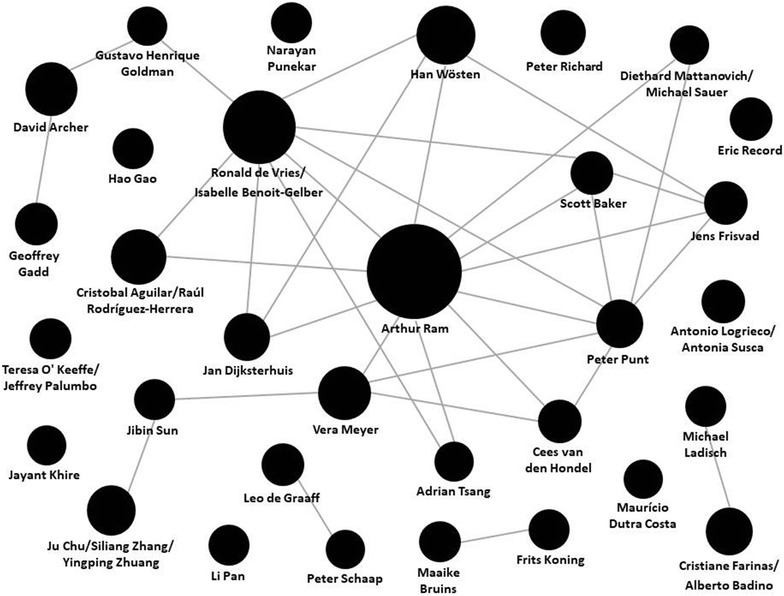
The *A. niger* research community is diverse and well-connected. Network of the community of researchers investigating *A. niger* biology or using the fungus as model organism based on our literature survey. Articles with ‘*Aspergillus niger*’ in title, abstract or keywords published in 2013–2017 were retrieved from PubMed. Scientists with at least 5 last (co-)authorships were selected (Additional file 3: Table 3). Size of circles roughly indicates number of published articles. Connections indicate collaborations as indicated by at least one co-authorship. Scientists are located mainly in Europe, followed by China, South America, North America, and India. The position of the circles is arbitrary. Please note that some connections/collaborations might be missing due to the stringency of our PubMed search

In an effort to map the landscape of international research groups currently working on *A. niger*, we retrieved all PubMed articles with ‘*Aspergillus niger*’ in title, abstract, and keywords published during the last 5 years (2013–2017), resulting in 2068 hits (Additional file 3: Table S3). Members of the community were defined by having authored at least 5 articles over the last 5 years. This list of researchers was then manually curated to highlight group leaders/PIs, and collaboration among research groups (as determined by at least one co-authorship). We focused on this relative short time span to ensure mapping of researchers actively working on *A. niger*, and included abstract and keywords in the search filter to expand the results to the community of those scientists not only studying *A. niger* biology, but also investigating the fungus in other relevant areas (e.g. bioremediation, geomicrobiology, pathogenicity, toxin production and food safety, agricultural microbiology). Based on previously reported information [91–93] we also compiled a list of (multinational) companies using *A. niger* as a workhorse for the production of citric acid and enzymes (Table 1). Our mapping of both basic and applied research on *A. niger* (Fig. 3, Table 1) shows that the community is diverse and geographically dispersed, yet (at least for the basic research community) well-connected.

## Future challenges for the *A. niger* community

**Fig. 4:**
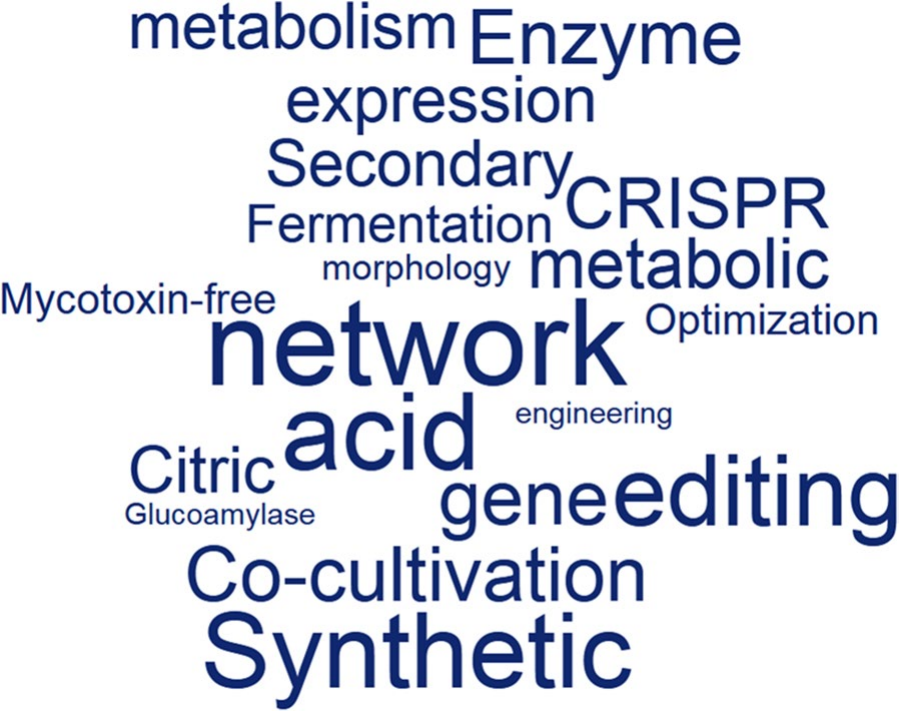
Predicting the next 20 years of *A. niger* research. Each author of this review independently predicted and independently weighted approximately 20 words that they hypothesize will commonly feature in future manuscript titles that also contain the words ‘*Aspergillus niger*’. The word cloud was generated online as described in Fig. 1

This historical overview has covered some of the scientific trends and key discoveries that have occurred in the field of *A. niger* biotechnology. Clearly, there are a diverse range of other industrially relevant fungi and bacteria that have also undergone revolutionary advances since their first use by early industrial microbiologists in the late nineteenth and early twentieth centuries. What does the future of industrial biotechnology hold for *A. niger*, and other microbial cell factories? In an attempt to answer this question, we generated predictions for common research themes and topics over the next 20 years of *A. niger* research (Fig. 4). This speculation indicated a hypothesized future focus on synthetic biology (including generation of mycotoxin-free isolates), network analysis (including genomics, gene expression and metabolomics), increased applications of co-cultivation technology and CRISPR-Cas9 genome editing, and a continued focus on secondary metabolism, fermentation, citric acid production, enzymes, and glucoamylase research (Fig. 4).

In general, three key components are becoming increasingly available in the biotechnologist’s toolbox; (1) publicly available and well-annotated genome data for several *A. niger* strains, and open-source bioinformatic pipelines that enable sophisticated comparative genomic and other analyses by non-coders; (2) guideline cultivation protocols with engineered macroscopic morphologies of *A. niger*, which facilitate improved product titres; (3) a versatile suite of molecular techniques for high-throughput gene functional analyses, including genome editing. What more has to come to fully understand and optimally exploit *A. niger*? In our opinion, the following outstanding questions need to be addressed by the community in the near future:

1. How can community efforts maintain and increase the quality and usability (from data deposition to analysis) of fungal datasets in light of the increasing amount of omics and literature data generated? How can we verify the accuracy of predictive algorithms to assign function to hypothetical genes?
2. How can we solve the “hypothetical protein problem”, and how can we assign function to those putative proteins? What is the best and easiest way to integrate genome, transcriptome, proteome, and metabolome data for powerful comparative omics approaches?
3. How can we generate accurate genome-wide metabolic networks that also are integrated with other omics data?
4. How can miniaturised cultivation in microtiter plate (or smaller) size be adapted for *A. niger* and other filamentous fungi for high-throughput screenings?
5. How can cell heterogeneity be investigated, so that variations in metabolism and gene-expression are not averaged over a whole colony or mycelium, and which new tools will foster these single-cell approaches?
6. How can stable and reproducible growth of *A. niger* in mixed cultures be achieved, both in academic research (e.g. to investigate activation of secondary metabolism) and industrial processes (e.g. for efficient enzyme production)? Which co-cultivation approaches and tools for the current paradigmatic shift in microbial cultivation [94] can be specifically adapted/developed for *A. niger* and other filamentous fungi?
7. Would concerted efforts to construct a genome-wide deletion and/or overexpression library of *A. niger*, similar to that existing for *S. cerevisiae* [95], *N. crassa* [96] and *Schizosaccharomyces pombe* [97], be sufficiently helpful for the community to warrant the substantial investment of research funds and resources?
8. How can a minimal *A. niger* genome be defined, and with which approach should it be generated? Which secondary metabolite clusters should be included or omitted?
9. Which synthetic biology tools to regulate different metabolic pathways in parallel can be developed or implemented, e.g. for the construction of genetic circuits to optimize metabolic fluxes for efficient production of enzymes, organic acids, or secondary metabolites?
10. Can *A. niger*, or other filamentous fungi, be exploited for “space biotechnological” purposes, as essential companions of astronauts for the autonomous production of food, enzymes, antibiotics, or for use in terraforming efforts?

## Conclusion

Given the tremendous advances in the knowledge of *A. niger* biology over the last century, and the concomitant development of bioinformatics, cultivation, and molecular tools which are now at disposal of the community, this industrial fungus has the potential to remain one of the most versatile fungal platform microorganism. *A. niger* offers a chassis for products which cannot be produced in easier to handle bacterial systems, and it is able to produce not only proteins and enzymes at high concentrations, but also pharmaceuticals which are beneficial for human and animal health. Indeed, we predict that *A. niger* will be one of the key organisms involved in the next industrial revolution: the change from a fossil-based economy to a bio-economy. At this pace, we are excited to witness what the future will bring.

## Additional files

**Additional file 1: Table S1**. Complete list of all studies retrieved from the PubMed database with 'Aspergillus niger' in the title from 1917–2017.

**Additional file 2: Table S2**. Number of studies for various filamentous fungi over the last 40 years.

**Additional file 3: Table S3**. Retrieved articles from the PubMed database with 'Aspergillus niger' in the title, abstract, or keywords over the last 5 years used to map the active research community.
